# Gait alterations during walking with partial body weight supported on a treadmill and over the ground

**DOI:** 10.1038/s41598-019-44652-y

**Published:** 2019-05-31

**Authors:** Ana Maria F. Barela, Gabriela L. Gama, Douglas V. Russo-Junior, Melissa L. Celestino, José A. Barela

**Affiliations:** 1 Institute of Physical Activity and Sport Sciences, Cruzeiro do Sul University, Rua Galvão Bueno, 868 São Paulo, SP Brazil; 2 Department of Physical Education, Institute of Biosciences, São Paulo State University, Av. 24-A, 1515 Rio Claro, SP Brazil

**Keywords:** Rehabilitation, Rehabilitation

## Abstract

Understanding the changes induced by body weight support (BWS) systems when non-disabled adults walk can help develop appropriate rehabilitation protocols. The purpose of this study was to investigate spatial-temporal gait alterations during walking with BWS on a treadmill and over the ground. Fourteen non-disabled young adults (including seven women) walked over the ground and on a treadmill with 0%, 10%, and 20% of BWS at 80% of their self-selected comfortable walking speed (baseline). The stride length and speed, step length, and stance and double-limb support durations were calculated and compared among the different conditions. The non-disabled adults modulated their spatial-temporal gait parameters according to the surface and percentage of BWS. They walked with shorter and slower strides and shorter steps and spent more time in contact with the support surface as they walked on the treadmill than as they did over the ground. Walking on the treadmill promoted less variability and a higher rate of change than did walking over the ground. Both the surface and amount of BWS should be taken into consideration when using BWS systems for (re)learning and/or reestablishing gait.

## Introduction

Partial body weight support (BWS) systems have been widely used as an alternative therapeutic strategy for gait training of different populations, including individuals with stroke^1–5^, Parkinson’s disease^6,7^, spinal cord injury^8^ and children with cerebral palsy^9–11^. Further, some researchers have examined the changes induced by BWS when non-disabled young adults walk in an attempt to develop an appropriate training protocol for those with gait impairment^12–17^. However, previous studies have investigated the use of BWS either on treadmills or over the ground^18^. To the best of our knowledge, only Barela *et al*.^14^ investigated gait with BWS on both surfaces in non-disabled adults, but were not able to control the walking speed when walking over the ground because of the incapability of the BWS system they used and the fact that only 30% of BWS was assessed.

As non-disabled adults walk on a treadmill using a BWS system, the cadence, duration of double-limb support, maximum extension of the hip, maximum flexion of the knee at toe-off, and hip flexion at initial contact decrease; conversely, the duration of single-limb support, dorsiflexion at initial contact, and maximum plantarflexion of the ankle at toe-off increase^17,19,20^. However, as young adults walk over the ground with BWS, the cadence, duration of double-limb support, maximum flexion and extension of the knee, maximum flexion of the hip, and maximum dorsiflexion of the ankle decrease, while the duration of single-limb support increases^13–15^. These results show that the use of a BWS system either on a treadmill or over the ground can modify the gait pattern of individuals with no gait impairment.

Among the different percentages of BWS for gait intervention, 30% of body weight unloading is the most employed for individuals with stroke on treadmills^21,22^. However, when walking over the ground with a BWS system, 30% of BWS does not seem to be the most appropriate percentage of body weight unloading mainly because it would decrease the generation of posterior muscle energy by the lower limb at the end of the terminal stance^23^. Thus, it is important to investigate the different aspects of gait patterns in non-disabled young adults walking with a body weight unloading of less than 30% of BWS on both treadmills and over the ground while controlling their walking speed to establish and implement better gait training protocols for those with gait impairment.

In the present investigation, we employed BWS on both walking surfaces (i.e., on a treadmill and over the ground) with similar speeds to examine the possible gait parameter changes depending on surfaces and/or body weight unloading in non-disabled young adults. First, we examined the spatio-temporal changes in non-disabled young adults walking on a treadmill and over the ground with different amounts of body weight unloading. We investigated whether the support surface on which the BWS system is employed and the amount of body weight unloading would modify gait patterns. Second, we examined gait variability during walking with different amounts of body weight unloading on both the surfaces. We investigated whether the surface on which the BWS system is employed and the amount of BWS would influence the variability in walking performance. Finally, we examined the rate of change between walking over the ground with no BWS at self-selected comfortable speed and walking with different amounts of body weight unloading on both the treadmill and over the ground. We investigated the amount of change between walking at a self-selected comfortable speed along a pathway and walking with different amounts of body weight unloading on the treadmill and over the ground.

## Methods

### Participants

Fourteen non-disabled young adults (seven men and seven women) were included in this study. Their mean (standard deviation [SD]) age, height, and mass were 26 (4.4) years, 1.73 (0.08) m, and 73.4 (14.4) kg, respectively. All participants reported no known injury, disease, or musculoskeletal disorder, which could alter their walking performance. They wore flat shoes during their participation in the study.

This study was conducted in accordance with the Declaration of Helsinki principles. The Institutional Ethics Committee of Cruzeiro do Sul University approved the study experimental protocol. An informed consent was obtained from all participants.

### Equipment and procedures

The spatio-temporal parameters of walking were assessed and registered using two inertial sensors (Physiolog 4 Silver 10D, Gait Up, S.A., Lausanne, Switzerland) placed on each foot of the participants for the entire session. The use of these sensors has been described previously, and they exhibit good accuracy and precision for gait analyses^24,25^. The participants were asked to walk at a self-selected comfortable speed along a 10-m walkway back and forth for five times in a total of 10 trials (“baseline”). The time they took to walk in each trial was recorded using two photocells (Cefise Biotecnologia Esportiva, Nova Odessa, SP, Brazil) placed 8 m apart along the walkway, and the mean walking speed was calculated. Thereafter, they walked with the assistance of two different BWS systems used in previous studies^5,26^ on a treadmill (TK35, Cefise Biotecnologia Esportiva, Nova Odessa, SP, Brazil) and over the ground (Finix Tecnologia, Cerquilho, SP, Brazil) with 0%, 10%, and 20% of BWS at approximately 80% of their baseline walking speed. This velocity percentage was used, as a previous investigation has revealed that non-disabled individuals walked 20% slower than their self-selected comfortable speed with the overground BWS system (Finix Tecnologia)^19^. The surface order (i.e., treadmill and over the ground) and the amount of body weight unloading were randomized among the participants. The speed of both the treadmill and the overground BWS systems was carefully controlled. The overground BWS system specifically contains a moving cart attached to the bottom of a suspended 7-m-long rail that allows backward and forward movements and is controlled by a belt system linked to a servomotor. A customized program (LabView, National Instruments, Corp., Austin, TX, United States) was developed to control the displacement, velocity, and acceleration of the moving cart.

Before evaluating the walking performance with each of the BWS systems, the percentage of body weight unloading was set according to the experimental condition (0%, 10%, and 20%), and the participants practiced for a few trials over the ground and for 1 min on the treadmill, providing similar amount of practice for familiarization with each of the six experimental conditions. Thereafter, the participants performed 10 trials over the ground and for 1 min on the treadmill under each experimental condition. All participants were instructed not to hold onto the handrails during treadmill walking.

### Data analyses

In this study, the spatio-temporal parameters selected for analyses were the stride length, stride speed, and percentage of the stance period duration, in which the measurements are conducted for a single lower limb, and the step length and percentage of the total double-limb support duration, in which the measurements are conducted for both lower limbs. Using the Gait Analysis Software PRO (Gait UP, S.A., Lausanne, Switzerland), the stride length, stride speed, stance period duration, and step length raw data were obtained. The percentage of double-limb support duration was calculated on the basis of the instants of heel strikes from each foot and the stance duration time; it was then converted into a percentage value, taking into consideration the stride duration.

For the overground conditions, data from the turns and first and last strides were discarded; for the treadmill conditions, data from the first 30 s and the last five strides were discarded. The means and SDs of all the spatio-temporal parameters were then calculated for each participant under each condition. Finally, the rate of change was calculated to examine the differences between walking with BWS (0%, 10%, and 20%) on each surface (over the ground and on a treadmill) and walking at a self-selected comfortable speed with no BWS system (baseline). The value of each stride under each experimental condition was divided by the mean value of the baseline.

### Statistical analyses

As there was no difference between the male and female participants and between sides, the data of all participants were pooled together, and those on the right and left sides were averaged together before making comparisons among the different experimental conditions. Multivariate analyses of variance (MANOVAs) for repeated measurements were employed using the surface (treadmill and over the ground) and BWS (0%, 10%, and 20%) as factors. The dependent variables were the stride length, stride speed, and percentage of stance duration in the first MANOVA and the step length and percentage of the total double-limb support duration in the second MANOVA.

The same procedures were adopted for comparing the mean, variability, and rate of change among the different experimental conditions (totaling six MANOVAs). When necessary, univariate analyses, Tukey Honest Significant Difference post hoc tests, and pairwise comparisons with Bonferroni adjustments were employed. An alpha level of 0.05 was adopted for the statistical tests, which were conducted using SPSS.

## Results

All participants could walk under all the experimental conditions. The mean walking speed at baseline and with the BWS systems was 1.28 (0.14) m/s and 1.03 (0.10) m/s, respectively. Table 1 lists the 95% confidence intervals of all gait parameters during 0%, 10%, and 20% of BWS on the treadmill and over the ground.

**Table 1:** The 95% confidence interval of the spatio-temporal gait parameters during 0%, 10%, and 20% of body weight support (BWS) on the treadmill and over the ground.

Variables	BWS	95% Confidence Interval					
		Mean		Variability		Rate of change	
		Treadmill	Overground	Treadmill	Overground	Treadmill	Overground
**Stride length (m)**							
	0%	0.85–0.93	1.18–1.27	0.026–0.036	0.053–0.077	0.61–0.68	0.86–0.91
	10%	0.83–0.91	1.18–1.27	0.027–0.038	0.060–0.118	0.60–0.66	0.85–0.91
	20%	0.82–0.91	1.15–1.24	0.026–0.042	0.073–0.109	0.59–0.66	0.83–0.90
**Stride speed (m/s)**							
	0%	0.61–0.67	1.01–1.12	0.017–0.024	0.074–0.107	0.49–0.52	0.81–0.86
	10%	0.61–0.67	1.01–1.13	0.016–0.026	0.078–0.148	0.49–0.52	0.81–0.87
	20%	0.61–0.67	1.00–1.12	0.020–0.031	0.086–0.148	0.49–0.52	0.80–0.86
**Stance duration (%)**							
	0%	63.9–66.6	61.6–63.8	1.884–2.494	1.949–2.360	1.06–1.10	1.02–1.05
	10%	64.0–66.6	58.6–62.0	1.570–2.377	2.134–3.446	1.06–1.10	0.96–0.99
	20%	63.4–66.1	58.0–60.7	1.715–2.714	2.417–3.737	1.05–1.09	0.96–1.00
**Step length (m)**							
	0%	0.43–0.47	0.59–0.63	0.023–0.031	0.036–0.047	0.62–0.68	0.85–0.90
	10%	0.42–0.46	0.59–0.63	0.020–0.036	0.042–0.074	0.61–0.67	0.85–0.91
	20%	0.41–0.46	0.57–0.62	0.023–0.039	0.045–0.069	0.59–0.67	0.82–0.84
**Double-limb support (%)**							
	0%	27.6–33.1	21.7–26.2	4.209–5.324	4.938–6.722	1.35–1.71	1.10–1.29
	10%	27.8–33.3	16.9–22.0	3.591–5.655	5.538–7.480	1.37–1.69	0.84–1.11
	20%	26.6–32.3	16.4–20.6	4.391–6.656	3.457–4.752	1.32–1.63	0.79–1.08

**Figure 1:**
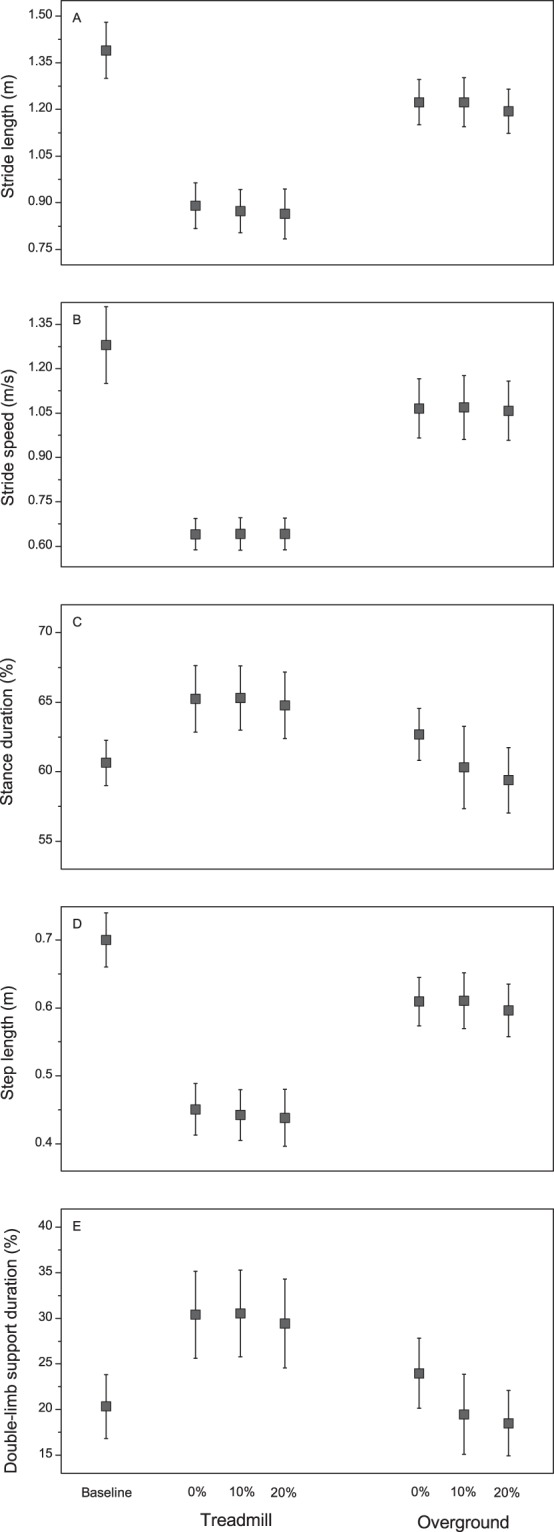
Mean and standard deviations of stride length (**A**), stride speed (**B**), percentage of stance duration (**C**), step length (**D**), and percentage of total double-limb support (**E**) at the baseline, 0%, 10%, and 20% of BWS on both treadmill and over the ground. Note: data from baseline are presented only as reference values.

Figure 1 depicts the mean (SD) values of all gait parameters during baseline and 0%, 10%, and 20% of BWS on both the treadmill and over the ground. The MANOVA for the stride length, stride speed, and stance duration revealed a main effect of the surface (Wilks’ Lambda = 0.01, F_3,11_ = 327.53, p < 0.01), BWS (Wilks’ Lambda = 0.23, F_6,48_ = 8.60, p < 0.01), and surface-BWS interaction (Wilks’ Lambda = 0.41, F_6,48_ = 4.56, p < 0.01). The univariate analyses revealed a surface effect for the stride length (F_1,13_ = 315.74, p < 0.01), stride speed (F_1,13_ = 1019.75, p < 0.01), and stance duration (F_1,13_ = 48.31, p < 0.01); BWS effect for the stride length (F_2,26_ = 4.034, p = 0.03) and stance duration (F_2,26_ = 23.45, p < 0.01); and surface-BWS interaction for the stance duration (F_2,26_ = 11.10, p < 0.01). The participants had shorter and slower strides and a longer stance duration when they walked with BWS on the treadmill than when they did over the ground (Fig. 1A–C). The pairwise comparisons revealed that the participants presented a longer stride length at 0% of BWS than at 20% of BWS (p = 0.04). The post hoc tests revealed that the participants presented longer stance durations at 0% and 10% of BWS than at 20% of BWS on both surfaces. Further, they presented a longer stance duration at 0% of BWS than at 10% over the ground.

The MANOVA for the step length and double-limb support duration revealed a main effect of the surface (Wilks’ Lambda = 0.04, F_2,12_ = 154.23, p < 0.01), BWS (Wilks’ Lambda = 0.31, F_4,50_ = 9.95, p < 0.01), and surface-BWS interaction (Wilks’ Lambda = 0.50, F_4,50_ = 5.16, p < 0.01). The univariate analyses revealed a surface effect for the step length (F_1,13_ = 303.99, p < 0.01) and double-limb support duration (F_1,13_ = 58.29, p < 0.01); BWS effect for the step length (F_2,26_ = 3.58, p = 0.04) and double-limb support duration (F_2,26_ = 21.10, p < 0.01); and surface-BWS interaction for the double-limb support duration (F_2,26_ = 10.50, p < 0.01). The participants had shorter steps and a longer double-limb support duration when they walked with BWS on the treadmill than when they did over the ground (Fig. 1D,E). The post hoc tests revealed that the participants presented a longer double-limb support duration at 0% of BWS than at 10% and 20% of BWS only when they walked over the ground.

**Figure 2:**
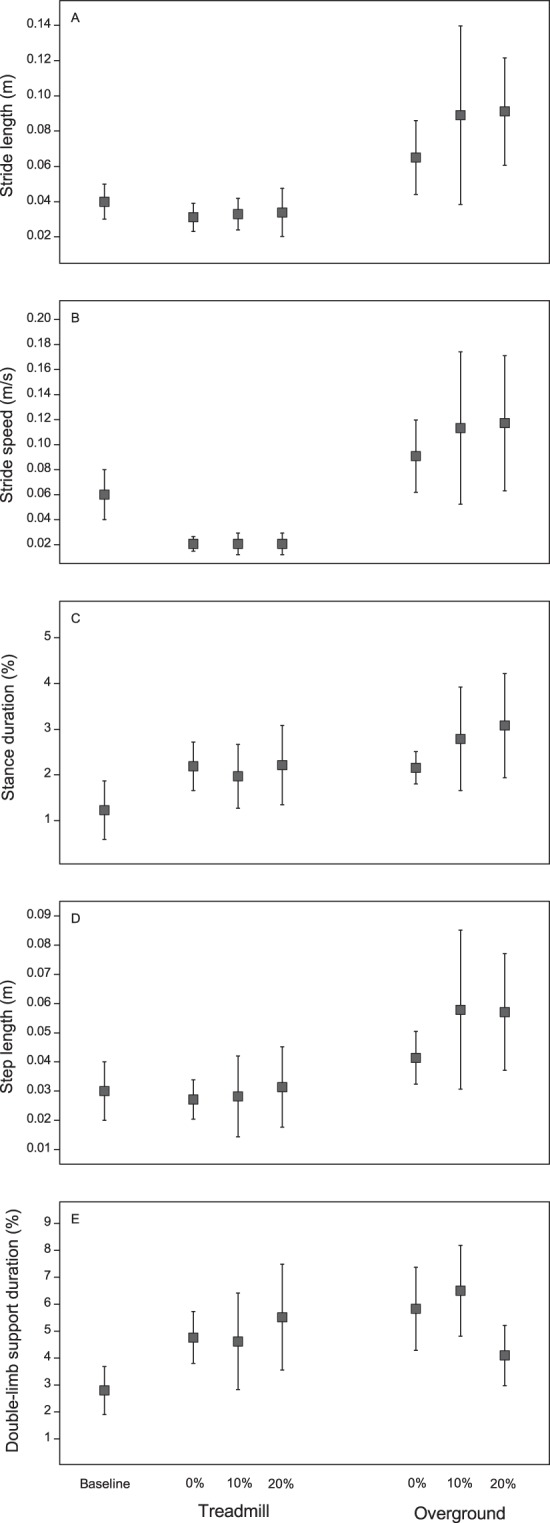
Mean and standard deviations for variability in stride length (**A**), stride speed (**B**), percentage of stance duration (**C**), step length (**D**), and percentage of total double-limb support (**E**) at the baseline, 0%, 10%, and 20% of BWS on both treadmill and over the ground. Note: data from baseline are presented only as reference values.

Figure 2 depicts the mean (SD) variability values of all the parameters during baseline and 0%, 10%, and 20% of BWS on both the treadmill and over the ground. The MANOVA for the stride length, stride speed, and stance duration revealed a main effect of the surface (Wilks’ Lambda = 0.15, F_3,11_ = 21.47, p < 0.01), but not of BWS (Wilks’ Lambda = 0.69, F_6,48_ = 1.60, p = 0.17) and surface-BWS interaction (Wilks’ Lambda = 0.68, F_6,48_ = 1.79, p = 0.14). The univariate analyses revealed a surface effect for the stride length (F_1,13_ = 59.85, p < 0.01), stride speed (F_1,13_ = 69.87, p < 0.01), and stance duration (F_1,13_ = 6.17, p = 0.03). The participants presented higher variabilities in their stride length, stride speed, and stance duration as they walked with BWS over the ground than as they did on the treadmill (Fig. 2A–C).

The MANOVA for the step length and double-limb support duration revealed a main effect of the surface (Wilks’ Lambda = 0.26, F_2,12_ = 16.74, p < 0.01), BWS (Wilks’ Lambda = 0.49, F_4,50_ = 5.45, p < 0.01), and surface-BWS interaction (Wilks’ Lambda = 0.29, F_4,50_ = 10.62, p < 0.01). The univariate analyses revealed a surface effect for the step length (F_1,13_ = 34.65, p < 0.01); BWS effect for the step length (F_2,26_ = 3.94, p = 0.03) and double-limb support duration (F_2,26_ = 4.38, p = 0.04); and surface-BWS interaction for the step length (F_2,26_ = 4.86, p = 0.03) and double-limb support duration (F_2,26_ = 18.99, p < 0.01). The post hoc tests revealed that the participants presented a higher variability in their step length as they walked over the ground than as they did on the treadmill, at 20% of BWS than at 0% of BWS when walking on the treadmill, and at 10% and 20% of BWS than at 0% of BWS when walking over the ground (Fig. 2D). They also presented a higher variability in their double-limb support duration as they walked over the ground than as they did on the treadmill and at 0% and 10% of BWS than at 10% of BWS when walking over the ground (Fig. 2E).

**Figure 3:**
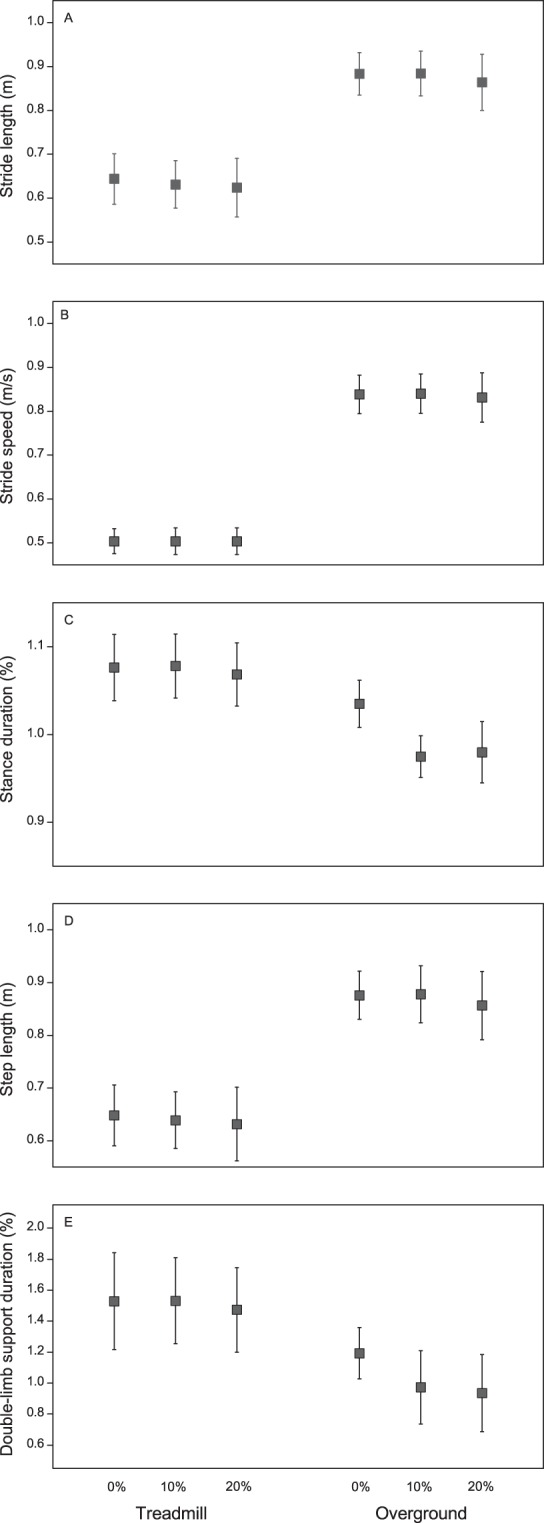
Mean and standard deviations for ratio of change in stride length (**A**), stride speed (**B**), percentage of stance duration (**C**), step length (**D**), and percentage of total double-limb support (**E**) at the baseline, 0%, 10%, and 20% of BWS on both treadmill and over the ground.

Figure 3 depicts the mean (SD) rate of change values of all the parameters during 0%, 10%, and 20% of BWS on both the treadmill and over the ground. The MANOVA for the stride length, stride speed, and stance duration revealed a main effect of the surface (Wilks’ Lambda = 0.004, F_3,11_ = 924.03, p < 0.01), BWS (Wilks’ Lambda = 0.22, F_6,48_ = 9.04, p < 0.01), and surface-BWS interaction (Wilks’ Lambda = 0.34, F_6,48_ = 5.83, p < 0.01). The univariate analyses revealed a surface effect for the stride length (F_1,13_ = 362.61, p < 0.01), stride speed (F_1,13_ = 3185.79, p < 0.01), and stance duration (F_1,13_ = 74.35, p < 0.01). In terms of the surface, the participants presented a higher rate of change in all parameters as they walked on the treadmill than as they did over the ground (Fig. 3A–C). In terms of the body weight unloading, the participants presented a higher rate of change in the stride length with 20% of BWS than with 0% of BWS on both the surfaces. For the stance duration, the post hoc tests revealed that the participants presented a higher rate of change with 10% of BWS than with 0% and 10% of BWS on the treadmill and with 10% and 20% of BWS than with 0% of BWS over the ground.

The MANOVA for the step length and double-limb support revealed a main effect of the surface (Wilks’ Lambda = 0.04, F_2,12_ = 167.26, p < 0.01), BWS (Wilks’ Lambda = 0.29, F_4,50_ = 10.78, p < 0.01), and surface-BWS interaction (Wilks’ Lambda = 0.56, F_4,50_ = 4.19, p < 0.01). The univariate analyses revealed a surface effect for the step length (F_1,13_ = 343.89, p < 0.01) and double-limb support duration (F_1,13_ = 55.83, p < 0.01); BWS effect for the step length (F_2,26_ = 3.51, p = 0.04) and double-limb support duration (F_2,26_ = 22.77, p < 0.01); and surface-BWS interaction for the double-limb support duration (F_2,26_ = 8.05, p < 0.01). The participants presented a higher rate of change in the step length and double-limb support duration when they walked with BWS on the treadmill than when they did over the ground (Fig. 3D,E). Finally, the post hoc tests revealed that the participants presented a higher rate of change in the double-limb support duration at 0% of BWS than at 10% and 20% of BWS over the ground.

## Discussion

The aim of this study was to investigate the spatio-temporal gait parameters of non-disabled young adults during walking with BWS on a treadmill and over the ground. The findings of this study can contribute to a better understanding on how BWS systems can modify gait patterns on both treadmills and over the ground.

Regarding the changes in the gait pattern according to the support surface and amount of BWS, the participants of this study voluntarily presented shorter and slower strides and shorter steps when they walked on the treadmill than when they did over the ground, although they were driven by an external input stimulating a similar walking speed. However, they spent a longer time in contact with the walking surface on the treadmill than over the ground. These results reveal that young adults could modulate time and space differently on each surface probably because the excursion of their center of mass was smaller when they walked on the treadmill than when they did over the ground^27^. These results could also be used to establish a strategy to maintain balance and stability during treadmill walking, which is a less stable surface than the ground^28^. However, at a higher percentage of BWS, the duration of their stance and double-limb support, mainly over the ground, decreased, which suggests that BWS can stimulate shorter periods of foot contact as it provides support for balance and consequently, walking stability^15,23^. These results corroborate those of previous studies^14,28^ and might represent a strategy to maintain the center of mass closer to the base of the support to maintain walking stability compared to the imbalance promoted by the treadmill’s belt backward movement^28^. Based on these results, it is important to know that if a treadmill is employed with BWS in clinical settings, strategies for stimulating longer and faster steps should be considered.

Regarding gait variability during walking on the treadmill and over the ground and the amount of BWS, the non-disabled young adults presented higher variabilities when they walked over the ground than when they did on the treadmill. Similar results have been found when comparing walking on a treadmill and over the ground with no BWS^29^; these suggest that walking over the ground with BWS allows more interaction among the degrees of freedom of body segments^30^. In terms of functionality, variability during gait practice is desirable and necessary for adaptability^31^. If more variabilities during practice are considered to lead to better retention and transfer to different contexts^32^ and consequently, motor learning^33,34^, the use of a BWS system over the ground would provide more benefits than that on a treadmill for (re)learning and/or reestablishing gait in individuals with gait impairment.

Finally, our study revealed that the non-disabled young adults presented a higher rate of change as they walked on the treadmill than as they did over the ground, even if the walking speed was controlled through a servo-motor in the overground BWS system and the treadmill’s belt. These results are particularly important if we consider that the main goal of a gait intervention program is to improve ambulation in daily life^35–37^. The lower the rate of change, the easier the generalization from intervention settings on a daily life basis. Therefore, walking over the ground with a BWS would promote better and more generalizable practice for individuals seeking to improve their walking capabilities in a clustered environment. This is an important issue that should be taken into consideration in any intervention protocol.

This study presents new insights related to the use of BWS on treadmill and over the ground; however, there are some limitations that should be considered. First, this study only examined spatio-temporal parameters, which might not account for all the changes in gait patterns with the use of BWS on different surfaces. However, we selected parameters related to the time and distance aspects of walking that constitute the performers’ stride characteristics. As such, these parameters represent their basic walking capability^38^. Future investigations should aim to include other measurements to provide a full description of gait pattern changes. Second, the participants walked at 80% of their self-selected walking speed with the BWS systems on both surfaces, which might have influenced their performance. However, we decided to employ similar walking speeds on both surfaces because a previous investigation revealed that the non-disabled young adults’ self-selected walking speed with BWS over the ground was 20% slower than their free self-selected walking speed^19^. Thus, the effects of manipulation of the walking speed and use of BWS on different surfaces remain to be investigated. Lastly, only a short period of familiarization was provided for the participants, which might not have been enough to reveal other effects of BWS and the surfaces during walking. Such a short period of familiarization was necessary owing to all manipulations employed in this study; this prevented any longer periods spent in the laboratory, although the period was similar for all participants in all experimental conditions. This issue still requires further investigation for a better understanding.

## Conclusion

The overall results of this study showed that the surface and the amount of body weight unloading modify gait patterns. The use of a BWS system over the ground seems to be more appropriate for gait intervention than that on a treadmill. However, future studies should investigate the effects on different populations with gait impairment.
